# Detailed characterization of the complete mitochondrial genome of the oceanic whitetip shark *Carcharhinus longimanus* (Poey, 1861)

**DOI:** 10.1007/s11033-024-09780-3

**Published:** 2024-07-19

**Authors:** Sadia A. Kamal, J. Antonio Baeza

**Affiliations:** 1 Department of Fisheries Biology and Genetics, Sher-e-Bangla Agricultural University, Dhaka, Bangladesh; 2 Department of Biological Sciences, Clemson University, Clemson, SC USA; 3 Smithsonian Marine Station at Fort Pierce, Smithsonian Institution, Fort Pierce, FL USA; 4 Departamento de Biología Marina, Universidad Catolica del Norte, Coquimbo, Chile

**Keywords:** Mitogenome assembly, Genomic resources, Purifying selection, Phylomitogenomics

## Abstract

**Background:**

The oceanic whitetip shark *Carcharhinus longimanus* (family Carcharhinidae) is one of the largest sharks inhabiting all tropical and subtropical oceanic regions. Due to their life history traits and mortality attributed to pelagic longline fishing practices, this species is experiencing substantial population decline. Currently, *C. longimanus* is considered by the IUCN Red List of Threatened Species as “vulnerable” throughout its range and “critically endangered” in the western north Atlantic. This study sequences and describes the complete mitochondrial genome of *C. longimanus* in detail.

**Methods and results:**

The mitochondrial genome of *C. longimanus* was assembled through next-generation sequencing and then analyzed using specialized bioinformatics tools. The circular, double-stranded AT-rich mitogenome of *C. longimanus* is 16,704 bp long and contains 22 tRNA genes, 2 rRNA genes, 13 protein coding genes and a 1,065 bp long control region (CR). Out of the 22 tRNA genes, only one (tRNA-Ser1) lacked a typical ‘cloverleaf’ secondary structure. The prevalence of TTA (Leu), ATT (Ile) and CTA (Leu) codons in the PCGs likely contributes to the AT-rich nature of this mitogenome. In the CR, ten microsatellites were detected but no tandem repeats were found. Stem-and-loop secondary structures were common along the entire length of the CR. Ka/Ks values estimated for all PCGs were < 1, indicating that all the PCGs experience purifying selection. A phylomitogenomic analysis based on translated PCGs confirms the sister relationship between *C. longimanus* and *C. obscurus*. The analysis did not support the monophyly of the genus *Carcharhinus*.

**Conclusions:**

The assembled mitochondrial genome of this pelagic shark can provide insight into the phylogenetic relationships in the genus *Carcharhinus* and aid conservation and management efforts in the Central Pacific Ocean.

**Supplementary Information:**

The online version contains supplementary material available at 10.1007/s11033-024-09780-3.

## Introduction

The Carcharhiniformes is the largest order of sharks consisting of 200 extant species, the majority of which belongs to the genus *Carcharhinus* in the family Carcharhinidae [1]. In this genus, the oceanic whitetip shark *Carcharhinus longimanus* is considered as the only true oceanic shark [2]. With a robust build and large rounded dorsal and long paddle-like pectoral fins, these sharks are further distinguished by the presence of white mottled markings on the tips of their pectoral, dorsal and tail fins, and black tips on their anal and ventral surface of pectoral fins [3, 4].

Oceanic whitetip sharks are epipelagic, found in shallow waters to at least 152 m deep [5, 6] in all tropical and subtropical ocean basins (above 20 °C) between 30° N and 35° S [2, 5]. Maximum body mass in this species can exceed 150 kg [7] and specimens can reach up to nearly 395 cm in total length (TL) [8, 9]. This highly migratory shark is reported to have a lifespan of about 17 years [8]. No major differences in growth rate have been observed between male and female oceanic whitetip sharks and sexual maturity is reached at 6–7 years in the two sexes [2]. Mating typically occurs in June and July, while parturition takes place between February and July [10]. Oceanic whitetip shark litter size ranges between 1 and 14, sharks are 55–75 cm in total length (TL) at birth, and reach maturity at approximately 170–200 cm TL [2]. A weak positive correlation exists between female size and litter size in *C. longimanus* [10]. *Carcharhinus longimanus* is recognized as one of the most prevalent top-level predators in open waters [2] playing a crucial role in maintaining the structure and function of coastal and marine ecosystems [11]. Their diet mostly consists of oceanic teleost fishes and cephalopods [2].

Oceanic whitetip sharks share many life history traits with other elasmobranchs (sharks, rays, and skates), including late sexual maturity, low fecundity, slow growth rate, as well as long gestation periods and lifespan [6, 11–14]. Given the aforementioned life-history traits, *Carcharhinus longimanus* is highly vulnerable to fishing pressure and is expected to experience prolonged recovery periods following population decline [9]. *Carcharhinus longimanus* is one of the most common bycatch species in tuna fisheries in offshore tropical waters [2] and has experienced major population decline during the last several decades [4]. This population decline is also due to their demand in the global shark fin trade [15–17]. Given the increasing fishing pressure and high catchability, the species is likely to experience a decline of more than 80% in population size within three generations’ time [5] Today, over 30% of all shark species face imminent risk of extinction primarily due to overfishing [18]. Despite the global implementation of no-retention policies for *C. longimanus* in tuna longline fisheries, this species remains highly vulnerable to longline fishing practices [9]. Considering all these factors, this species, which was previously labeled as ‘vulnerable’ (VU) [5], is now categorized as ‘critically endangered’ (CR) by the IUCN [19], raise concerns of their conservation and management status [20].

Several studies on the biology and ecology of this imperiled shark have been conducted [2, 5, 9, 10, 16, 19], but very few genetic and genomic resources exist in this and other congeneric species of conservation concern. Studies based on short mitochondrial gene markers (CR) have found low levels of genetic diversity in the Indian and Atlantic Oceans, and restricted gene flow between the western and eastern Atlantic Ocean [6]. In a more recent study, using the entire mitochondrial DNA CR, a segment of the mitochondrial PCG *nad4*, and 12 nuclear microsatellite loci, weak but statistically significant differentiation was reported between the Western Atlantic and Indo-Pacific Oceans, with additional significant matrilineal structure between Indian and Pacific Oceans but no population structure within the Western Atlantic [21].

In this study, we have sequenced, assembled, and described in detail the complete mitochondrial genome of the Oceanic Whitetip Shark *Carcharhinus longimanus*. Following the protocols in Baeza [22], we analyzed nucleotide composition of the entire mitochondrial genome as well as codon usage profiles of and selective constraints in protein coding genes. We also explored the secondary structure of each identified tRNA gene and investigated the architecture of the control region (CR). We note that Li [23] did sequence the mitochondrial genome of *C. longimanus* from the South China Sea. However, this previous study did not characterize the mitochondrial genome of the species in detail as we have done here. By characterizing the complete mitochondrial genome of the Oceanic Whitetip shark, *C. longimanus*, we are aiming to support management and conservation strategies in this critically endangered shark.

## Methods

To assemble the mitochondrial genome of the Oceanic Whitetip Shark *Carcharhinus longimanus*, we extracted genomic DNA (gDNA) from a specimen (SIO:1734e3c7-b223-40cc-b5eb-72d3b23579eb) deposited at the fish collection of the National Museum of Natural History, Smithsonian Institution, Washington DC, USA. The specimen was collected in the tropical Central Pacific Ocean (07° 45.0ʹ N, 141° 47.0ʹ W), southeast of Hawaii, on September 9, 1997, while onboard the RV Townsend Cromwell. gDNA was extracted from muscle tissue using an AutoGenPrep 965 automated DNA extraction robot (AutoGen, Holliston, MA, USA) according to the manufacturer’s guidelines. Next, an Illumina library was prepared following the standard NEB Ultra II DNA library prep kit (New England Biolabs, Ipswich, MA, USA) protocol. The library was sequenced on an Illumina NovaSeq (Illumina, San Diego, CA, USA) using a 2 × 150 cycle sequencing strategy. A total of 8,521,356 pairs of reads were employed to assemble ‘de novo’ the mitochondrial genome of *Carcharhinus longimanus* using the pipeline GetOrganelle v. 1.6.4 [24]. For the assembly, we used as a seed the mitochondrial genome of the congeneric *C. falciformis*, available in NCBI’s GenBank (accession number: OM885432). The run used k-mer sizes of 21, 55, 85, and 115. The sequence data are part of a project to sequence mitochondrial genomes of marine fishes occurring in the Exclusive Economic Zone of the United States based on voucher specimens (BioProject: PRJNA720393) and data are deposited on GenBank (BioSample: SAMN31811566).

The assembled genome of *C. longimanus* was first annotated using the webserver MITOS2 (https://mitos2.bioinf.uni-leipzig.de/—[25]) and the nucleotide composition of the whole mitochondrial genome was analyzed using the software MEGAX [26]. This first in silico annotation was manually curated using the web server Expasy (https://web.expasy.org/—[27]) in order to correct the start and stop codons of the protein coding genes. The entire mitochondrial genome was visualized using the web server Chloroplot (https://irscope.shinyapps.io/Chloroplot/—[28]). The transfer RNA genes (tRNA) were identified using the software MiTFi [29] as implemented in the web server MITOS2 and the secondary structure of each tRNA was visualized using the web server Forna (http://rna.tbi.univie.ac.at/forna/—[30]). The number and frequency of each codon in all protein codon genes was estimated using the vertebrate mitochondrial code in the web server Sequence Manipulation Suite (SMS) (https://www.bioinformatics.org/sms2/—[31]). Relative synonymous codon usage (RSCU), defined as the ratio of the observed frequency of codons to the expected frequency, of all concatenated protein coding genes was estimated and visualized using the EZcodon tool in the web server EZmito (https://ezmito.unisi.it/ezcodon—[32]). MEGAX was also used to analyze the nucleotide composition of ribosomal RNA (rRNA). Selective pressures acting on each mitochondrial PCG were examined while estimating rates of non-synonymous substitutions per non-synonymous site (Ka), synonymous substitutions per synonymous site (Ks) and the Ka/Ks ratio (ω) for each PCG using the program KaKs_calculator Toolbox 2.0 [33] with *C. leucas* (KF646785) as the outgroup. PCGs with Ka/Ks values below 1 experience negative (purifying) selection, whereas values above 1 indicate positive (diversifying) selection [33].

The long, non-coding control region (CR) was studied in detail. Repeats within the region were found using the BioPHP Microsatellite Repeats Finder web server (http://insilico.ehu.es/mini_tools/microsatellites/—[34]) and the Tandem Repeat Finder: 4.09 Version web server (https://tandem.bu.edu/trf/trf.basic.submit.html—[35]). Predictions of secondary structure of these regions were provided by RNAfold web server (http://rna.tbi.univie.ac.at/cgi-bin/RNAWebSuite/RNAfold.cgi—[36]) to observe the presence of hairpin and loop structures. While RNAfold calculates minimum free energy (MFE) and relies on experimental data for scoring parameters, it fails to identify unconventional RNA structures arising from tandem repeats in mitochondrial genomes [37]. To overcome this limitation, we opted to explore the secondary structure of the same region using the hybrid method MXFold2 (http://ws.sato-lab.org/mxfold2/—[37]) which provides a more accurate prediction by incorporating folding scores obtained from deep-neural network trained on extensive data and avoids overfitting by using thermodynamic parameters to evaluate previously unobserved structures.

### Phylomitogenomics of the genus *Carcharhinus*

To reveal the phylogenetic position of *C. longimanus* within the genus *Carcharhinus*, the newly assembled mitochondrial genome together with other 18 mitogenomes available in GenBank belonging to congeneric species were used for maximum likelihood (ML) phylogenetic inference. Given the evolutionary history of the genus *Carcharhinus*, which has been found to display incomplete lineage sorting and polytomies in previous phylogenetic inferences due to rapid radiation of its lineages [38–41], we carefully considered the phylogenetic pipeline used in this study. To address the previously reported phylogenetic complexities in the genus, dividing the alignment into gene partitions and selecting evolutionary models for each gene is necessary. Therefore, we decided to employ an ML analysis using a translated alignment of the protein coding genes.

A total of 19 other mitochondrial genomes were used in this study, including representatives from various genera of the family Carcharhinidae such as *Galeocerdo* (n = 2 species, *G. cuvier*), *Glyphis* (n = 5 species), *Lamiopsis* (n = 2 species), *Loxodon* (n = 2 species), *Rhizoprionodon* (n = 1 species), *Scoliodon* (n = 3 species), *Triaenodon* (n = 3 species, *T. obesus*), and the blue shark *Prionace glauca* and lemon shark *Negaprion brevirostris*. Additionally, mitochondrial genomes from closely related families Scyliorhinidae (belonging to the genera *Cephaloscyllium* (n = 2 species), *Scyliorhinus* (n = 2 species), and *Poroderma pantherinum*), Triakidae (n = 4 species, *Hemitriakis japonica* + *Mustelus* spp.), and Pentanchidae (*Galeus melastomus*, *Halaelurus buergeri*, and *Parmaturus melanobranchus*) were used as outgroups.

The analysis proceeded in a manner identical to that detailed in Baeza [22]. We first extracted all 13 PCG nucleotide sequences from all mitochondrial genomes and translated them to amino acids using the programs MEGA X and Clustal Omega [42], respectively. Poorly aligned regions in each PCG alignment were removed with trimAl [43] and best fitting models of sequence evolution for each PCG selected with ProtTest [44]. The best model selected was mtMAM + I + G4, applied to each of 13 partitions (one per PCG). Lastly, the concatenated and partitioned PCG amino acid dataset was used to perform a ML analysis in the program IQ-TREE version 1.6.10 using the default options [45]. The robustness of the ML tree topology was assessed by 1,000 bootstrap (method: UFboot) iterations of the observed dataset.

## Results and discussion

### Mitochondrial genome assembly and description

**Fig. 1:**
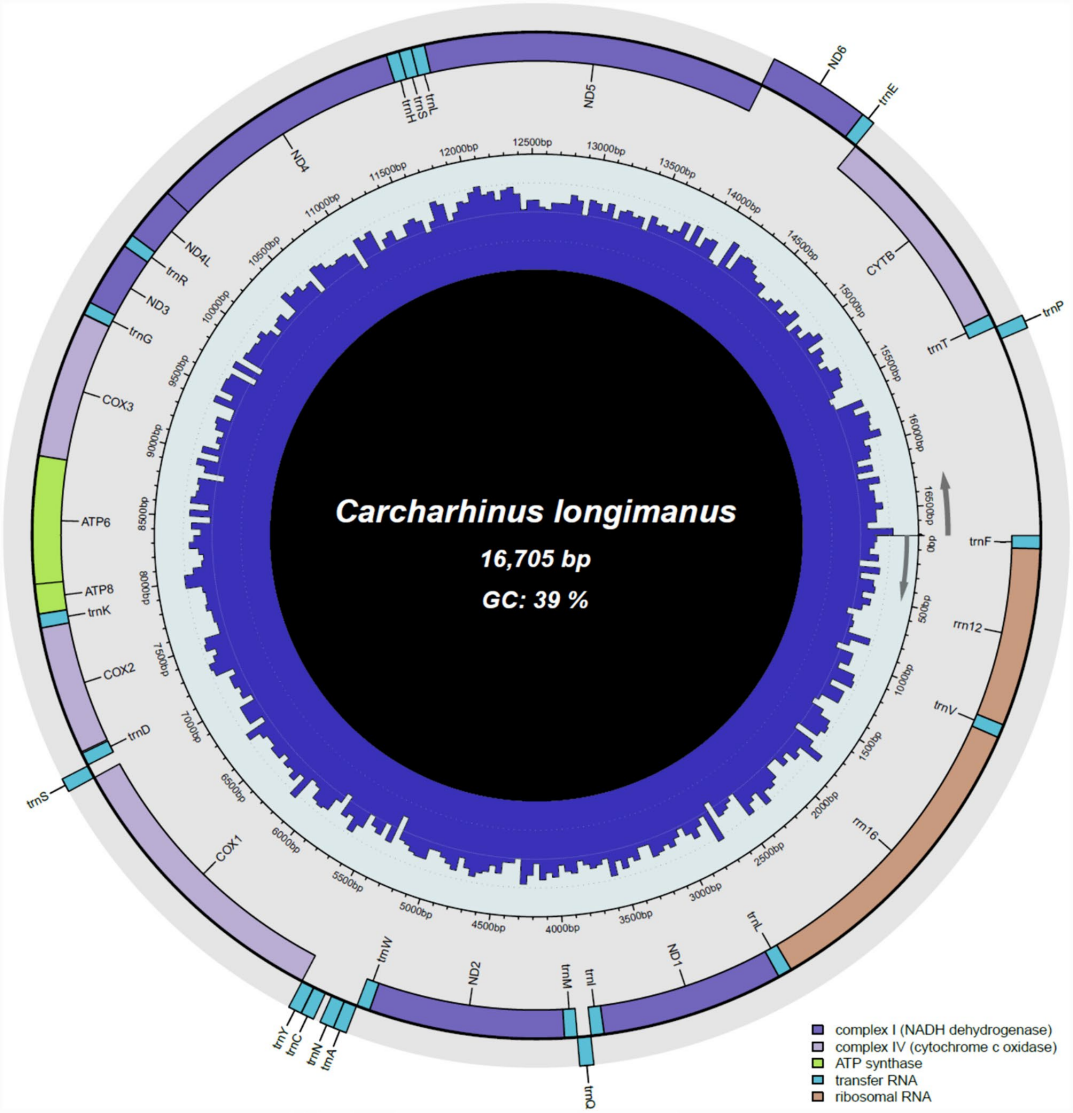
Circular DNA mitochondrial genome map of *Carcharhinus longimanus*. The annotated map depicts 22 transfer RNA (tRNA) genes, 13 protein-coding genes (PCGs), 2 ribosomal RNA genes (rrnS: 12S ribosomal RNA and rrnL: 16S ribosomal RNA), and a putative control region

The pipeline GetOrganelle assembled a complete mitochondrial genome of *Carcharhinus longimanus* (OP057117) with an average coverage of 12.6 × and 51.6 × per k-mer and base, respectively. The mitochondrial genome of *C. longimanus* is 16,705 bp in length and contains 22 tRNA genes, 2 rRNA genes, 13 protein coding genes and a non-coding control region (CR) (Fig. 1; Table 1). Most genes reside on the heavy, positive strand of the genome, while *nad6* and eight tRNAs (tRNA-Gln, tRNA-Asp, tRNA-Ala, tRNA-Cys, tRNA-Tyr, tRNA-Ser2, tRNA-Pro, and tRNA-Glu) are located on the light, negative strand. Other *Carcharhinus* spp. exhibit similar mitochondrial genome lengths ranging between 16,701 bp (*C. falciformis*—[46], *C. macloti*—[47] and 16,719 bp (*C. acronotus*—[47]). The mitochondrial gene order herein described for *C. longimanus* is identical to that documented before for other congeneric and cofamilial species [46–50].

**Table 1:** Mitochondrial genome of *Carcharhinus longimanus*

Name	Type	Start	Stop	Strand	Length (bp)	Start	Stop	Anticodon	Continuity
trnF(gaa)	tRNA	1	70	+	70			GAA	1
rrnS	rRNA	72	1029	+	958				-3
trnV(tac)	tRNA	1027	1098	+	72			TAC	23
rrnL	rRNA	1122	2768	+	1647				-1
trnL2(taa)	tRNA	2768	2842	+	75			TAA	0
nad1	PCG	2843	3817	+	975	TAA	ATG		0
trnI(gat)	tRNA	3818	3887	+	70			GAT	1
trnQ(ttg)	tRNA	3889	3960	−	72			TTG	-1
trnM(cat)	tRNA	3960	4028	+	69			CAT	0
nad2	PCG	4029	5075	+	1047	ATG	TAG		-2
trnW(tca)	tRNA	5074	5144	+	71			TCA	1
trnA(tgc)	tRNA	5146	5214	−	69			TGC	0
trnN(gtt)	tRNA	5215	5287	−	73			GTT	5
OL		5293	5322	+	30				0
trnC(gca)	tRNA	5323	5390	−	68			GCA	1
trnY(gta)	tRNA	5392	5460	−	69			GTA	1
cox1	PCG	5462	7018	+	1557	GTG	TAA		0
trnS2(tga)	tRNA	7019	7089	−	71			TGA	3
trnD(gtc)	tRNA	7093	7162	+	70			GTC	7
cox2	PCG	7170	7860	+	691	ATG	T(AA)		0
trnK(ttt)	tRNA	7861	7934	+	74			TTT	1
atp8	PCG	7936	8103	+	168	ATG	TAA		-10
atp6	PCG	8094	8777	+	684	ATG	TAA		-1
cox3	PCG	8777	9562	+	786	ATG	TAA		2
trnG(tcc)	tRNA	9565	9634	+	70			TCC	0
nad3	PCG	9635	9985	+	351	ATG	TAG		-2
trnR(tcg)	tRNA	9984	10,053	+	70			TCG	0
nad4l	PCG	10,054	10,350	+	297	ATG	TAA		7
nad4	PCG	10,344	11,724	+	1381	ATG	T(AA)		0
trnH(gtg)	tRNA	11,725	11,793	+	69			GTG	0
trnS1(gct)	tRNA	11,794	11,860	+	67			GCT	0
trnL1(tag)	tRNA	11,861	11,932	+	72			TAG	0
nad5	PCG	11,933	13,762	+	1830	ATG	TAA		-5
nad6	PCG	13,758	14,279	−	522	ATG	AGG		0
trnE(ttc)	tRNA	14,280	14,349	−	70			TTC	2
cob	PCG	14,352	15,497	+	1146	ATG	TAG		-1
trnT(tgt)	tRNA	15,497	15,568	+	72			TGT	2
trnP(tgg)	tRNA	15,571	15,639	−	69			TGG	33
CR		15,673	16,704	+	1032				1

Arrangement and annotation

The nucleotide composition of the studied mitochondrial genome is: A = 31.5%, T = 30.1%, G = 13.1%, C = 25.3%, with a high A + T content (61.5%) similar to that reported before for other congeneric and cofamilial species. In the genus *Carcharhinus*, A + T content has been reported to range between 59.9% in the Silky shark *Carcharhinus falciformis* [46] and 62.57% in the Bull shark *Carcharhinus leucas* [42]. In the order Carcharhiniformes, the lowest and highest reported A + T composition is 52.86% in the blotchy swellshark *Cephaloscyllium umbratile* [47] and 63.62% in the false catshark *Pseudotriakis microdon* [47], respectively. The high mutation rate from G to A in mitochondrial DNA may explain, in part, the A + T rich nature of this and other related mitochondrial genomes [51].

The PCGs of *C. longimanus* contain 3811 codons and range in length between 168 bp (*atp8*) and 1,830 bp (*nad5*) (Table 1). Start codons included ATG in 12 different PCGs and GTG in *cox1* (Table 1). The stop codon TAA was used in nine different PCGs, TAG was used in *cob, nad2* and *nad3*, and AGG was used in *nad6*. The incomplete termination codon T was used in *cox2* and *nad4*. This occurrence of incomplete stop codons is common in mitochondrial PCGs of eumetazoans, including sharks [52].

**Fig. 2:**
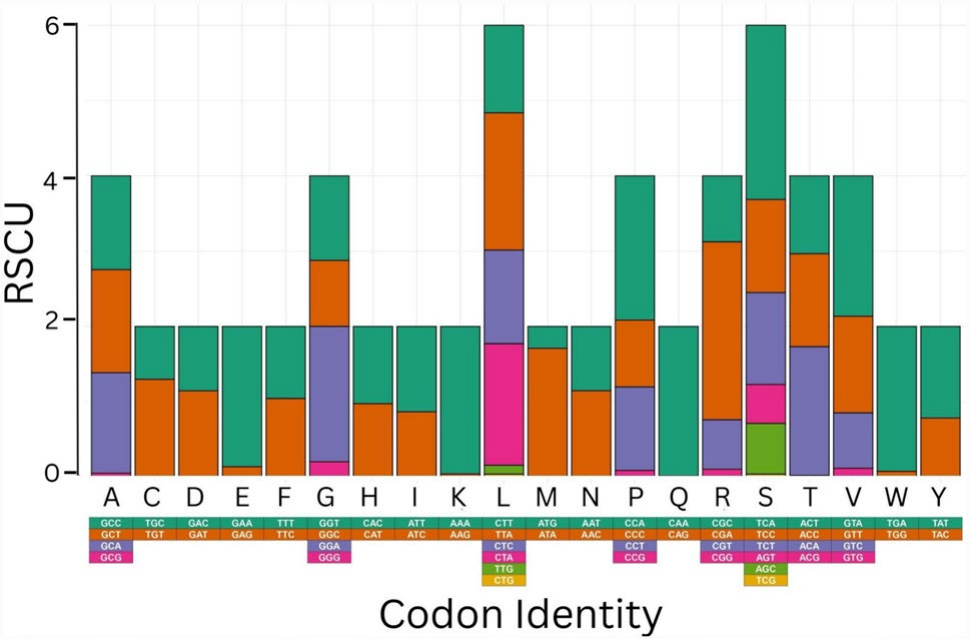
Codon usage analysis of PCGs in the mitochondrial genome of *Carcharhinus longimanus*. All 20 amino acids-alanine (A), cysteine (C), aspartic acid (D), glutamic acid (E), phenylalanine (F), glycine (G), histidine (H), isoleucine (I), lysine (K), leucine (L), methionine (M), asparagine (N), proline (P), glutamine (Q), arginine (R), serine (S), threonine (T), valine (V), tryptophan (W), tyrosine (Y) are listed by their one-letter abbreviations along the horizontal axis

In the mitochondrial PCGs of the species under study, there is bias in codon usage. Excluding start and stop codons, the most frequently used codons in the PCGs of the examined species were TTA (Leu), used 202 times (5.3%), followed by ATT (Ile), used 199 times (5.22%); and CTA (Leu), used 179 times (4.69%) (Online Resource 1). The least frequently used codons were CAG (Gln), used once (0.026%) followed by CGG (Arg), TCG (Ser) and ACG (Thr) each used twice at 0.052%. Relative synonymous codon usage (RSCU) analysis of PCGs in *C. longimanus* revealed that codons encoding Alanine, Serine, Leucine, Threonine, Glycine, Arginine and Proline are the most frequently used, whereas codons coding for Asparagine, Glutamine, Cysteine and Lysine were rare (Fig. 2). Codons starting with A or T are commonly used in comparison to other synonymous codons, for example, the codon for glutamine CAG was rare, which is consistent with previous observations of shark species in the order Carcharhiniformes, including the congeneric *Carcharhinus acronotus* [47].

In *C. longimanus*, all 13 mitochondrial PCGs exhibited Ka/Ks ratios < 1, indicating that these genes are exposed to ‘negative’ (= purifying) selection. The *cox2* gene featured the highest Ka/Ks ratio (Ka/Ks = 0.508, P = 0.066) while *nad1* exhibited the lowest Ka/Ks ratio (Ka/Ks = 0.007, P = 3.78E−45) (Table 2). Previous research has confirmed that mitochondrial PCGs exhibit higher mutation rates compared to nuclear genes [53]. Consistent with this notion, our results reveal a prevalence of PCGs undergoing negative selection, which is expected to eliminate deleterious mutations, rather than diversifying selection. This gradual accumulation of mutations over time suggests that negative or purifying selection is substantial in the evolution of mitogenomes [54]. Previous studies describing the mitochondrial genome of congeneric sharks have not explored selective pressures in PCGs. However, purifying selection affecting all 13 mitochondrial PCGs have been reported before in sharks belonging to the families Scyliorhinidae (i.e., *Cephalloscyllium umbratile* and *Scyliorhinus canicula*) and Proscylliidae (i.e., *Proscyllium habereri*—[43]), among others (e.g., in the Lemon shark *Negaprion brevirostris*—[22]).

**Table 2:** Selective pressure analysis in the protein coding genes (PCGs) of *Carcharhinus longimanus* indicating purifying selection in all genes with Ka/Ks < 1

Sequence	Ka	Ks	Ka/Ks	P-value
nad1	0.004086	0.548578	0.007448	3.78E−45
nad2	0.00774	0.678907	0.011401	2.23E−52
nad3	0.008117	0.4696	0.017285	6.55E−15
nad4	0.0333873	0.232658	0.143504	4.27E−20
nad5	0.0143924	0.686124	0.0209764	3.53E−77
nad6	0.012766	0.476056	0.0268161	4.93E−18
nad4l	0.013756	0.593875	0.023162	5.38E−13
cob	0.013866	0.402372	0.03446	1.63E−36
atp6	0.005916	0.422197	0.014012	1.26E−26
atp8	0.016093	0.500994	0.032121	1.17E−06
cox1	0.003239	0.335226	0.009663	6.16E−46
cox2	0.003565	0.231419	0.015405	2.06E−16
cox3	0.004864	0.361279	0.013463	2.81E−25

**Fig. 3:**
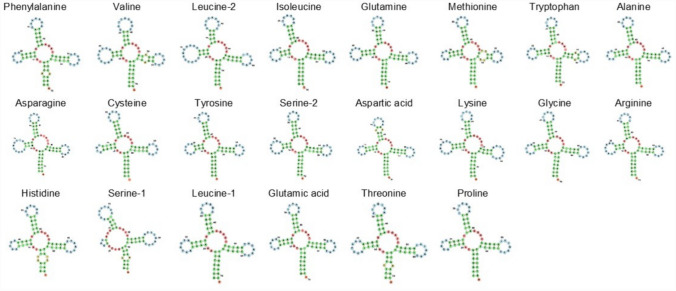
Secondary structure of tRNAs in the mitochondrial genome of *Carcharhinus longimanus*

In the mitochondrial genome of C. *longimanus*, the length of the tRNA genes ranged between 67 bp (tRNA-Ser1) and 75 bp (tRNA-Leu2) and all of them but one exhibited a typical ‘cloverleaf’ secondary structure (Fig. 3). The software MITFI predicted that the tRNA-Ser1 gene was missing the dihydrouridine loop. Our observations coincide with that observed in most representatives of the genus *Carcharhinus* in which all tRNA genes exhibit a cloverleaf secondary structure except tRNA-Ser1 that is truncated (i.e., *C. albimarginatus*—[55], *C. amblyrhynchoides*—[48], *C. perezi*—[56], *C. brachyurus*—[49], *C. limbatus*—[50]). Interestingly, *C. amblyrhynchoides* [57] and *C. melanopterus* [58] exhibit a truncated tRNA-Ser2 (lacking the D-arm) instead of tRNA-Ser1. While the cloverleaf shape of tRNA molecules generally contributes to the overall stability of their tertiary structure, a deviation from the typical cloverleaf structure, particularly in the tRNA-Ser1 gene, is commonly observed in almost all mitochondrial genomes of eumetazoans [25, 59].

In *C. longimanus*, the two ribosomal RNA (rRNA) genes are found in the positive strand. The 16S rRNA, 1,647 bp long, is located between tRNA-Va l and tRNA-Leu2, while 12S rRNA, 958 bp long, is located between tRNA-Val and tRNA-Phe. The nucleotide composition estimated for 16S rRNA is A = 36.06%, T = 26.59%, G = 16.69% and C = 20.64% and for 12S rRNA is A = 33.82%, T = 24%, G = 18.68% and C = 23.48%. The two rRNA genes are AT-rich in line to that reported for other congeneric species, including the Blacktip reef shark *Carcharhinus melanopterus* and the Bull shark *Carcharhinus leucas* [47].

The 1,065 bp long putative CR is located between the genes tRNA-Pro and tRNA-Phe. The nucleotide composition of the CR is A = 31.3%, T = 35.3%, C = 19.9%, and G = 13.5%, which is within range reported for the CR of other congeneric sharks [46, 47, 50]. In the genus *Carcharhinus*, the lowest and highest A + T content reported for the CR is 66.16% in the Blacktip reef shark *C. leucas* and 68.2% in the Hardnose shark *C. macloti*, respectively [47]. Stem-loop structures as well as microsatellite repeats were found within the CR. The web server microsatellite repeats-finder reported 10 microsatellites along this region, most of them AT-rich, repeated between 2 and a maximum of 5 times (Online Resource 2). Most microsatellites contain AA or TT dinucleotide repeats, with few CC and TA dinucleotides found towards the 3ʹ-end of the CR. The Tandem Repeats finder web server did not detect any repeats in the control region of *C. longimanus*. However, in the family Triakidae, instances of tandem repeats have been identified in the CR of species belonging to the genera *Galeorhinus*, *Triakis* and *Mustelus* (*G. galeus, T. megalopterus M. palumbes, M. asterias* and *M. mosis*) [41], but no tandem repeats have been reported in *M. canis* and *M. norrisi* [60]. Also, the RNAfold web server determined two possible secondary structures for the control region (Online Resource 3). Predicted Gibbs free energy (ΔG) values for the optimal and centroid RNA predicted secondary structures were ΔG = − 202.10 kcal/mol and ΔG = − 162.14 kcal/mol, respectively. According to this analysis, numerous stem-and-loop structures of different sizes were distributed along the entire CR. The MXFold2 web server predicted a more accurate analysis of the secondary structure of CR that also included multiple stem-and-loop structure (Online Resource 4). No previous study has analyzed in detail the CR of congeneric sharks. However, microsatellites are commonly observed in the CR of other sharks and the predicted secondary structure invariably exhibit stem-and-loops, as reported before in the closely related sharks *Galeus melastomus, Negaprion brevirostris*, *Odontaspis ferox,* and *Prionace glauca* [22, 52] and other distantly related species (i.e., the Grey bamboo shark *Chiloscyllium griseum*—[61]). The control region exhibits higher evolutionary rate compared to the other mitochondrial regions, making it an ideal tool for studying genetic diversity and population structure in representatives of the family Carcharhinidae.

### Phylomitogenomics of the genus *Carcharhinus*

**Fig. 4:**
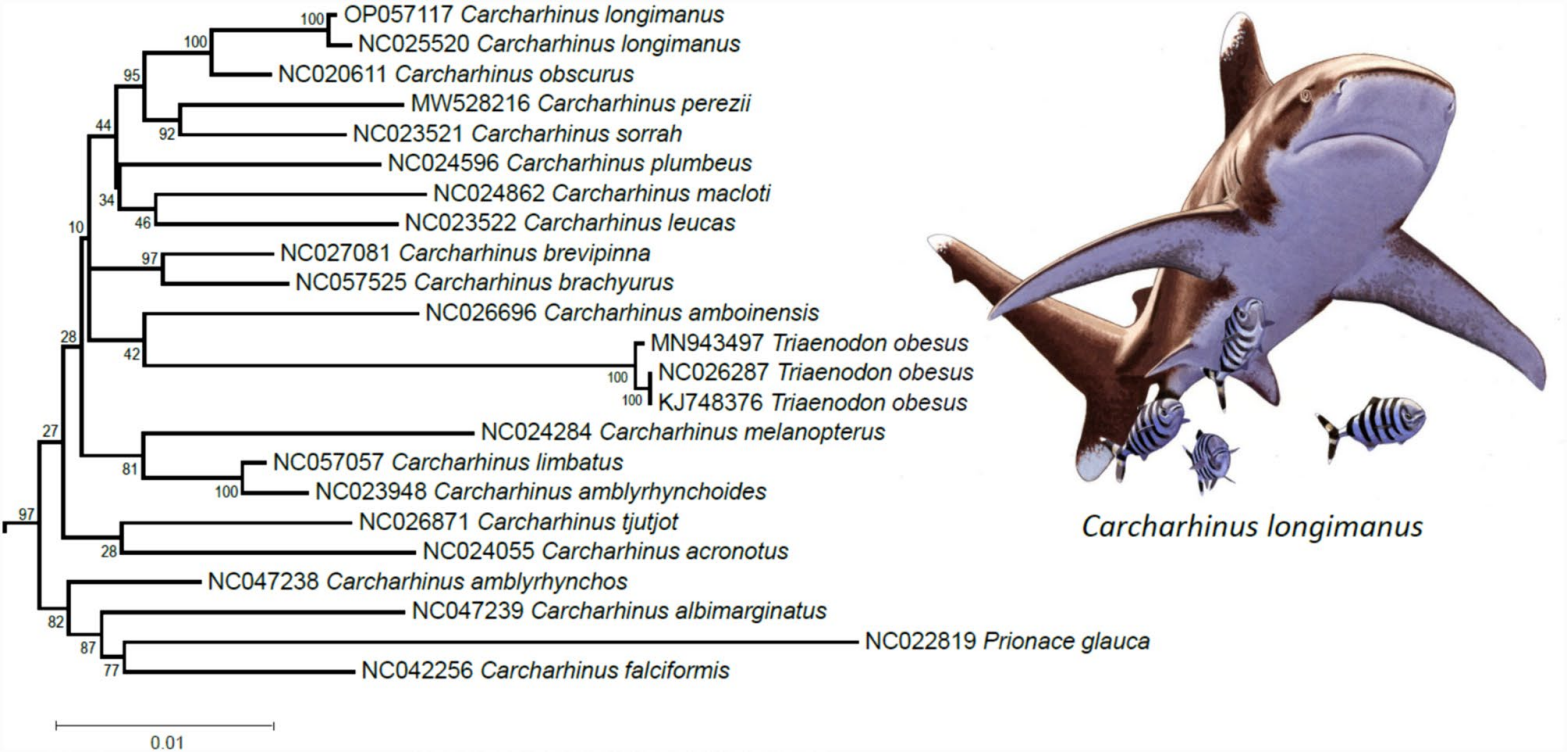
Total evidence phylogenetic tree obtained from ML analysis based on a concatenated alignment of amino acids of the 13 protein-coding genes present in the mitochondrial genome of the Oceanic Whitetip shark *Carcharhinus longimanus* and other representatives of the genus *Carcharhinus* and family Carcharhinidae. The robustness of the ML tree topology was ascertained by 1000 bootstrap pseudoreplicates (numbers above or below the nodes) of the tree search. Depiction of *C. longimanus* by Kókay Szabolcs (used with permission)

Our ML analysis (51 terminals, 3,799 amino acid characters, 737 informative sites) did not support the monophyly of the genus *Carcharhinus* (Fig. 4) due to the position of the blue shark *Prionace glauca* and the genus *Trianodon*, represented by 3 mitochondrial genomes belonging to the same species, *T. obesus*, in our study, which nested deep within a well-supported clade (bootstrap support value [bv] = 93) composed of all sharks belonging to the genus *Carcharhinus* used in the phylogenetic analysis. Specifically, *T. obesus*, formed a moderately supported (bv = 72) clade with *C. amboinensis* and *C. melanopterus* within a larger well supported clade (bv = 93) containing all other representatives of the genus *Carcharhinus* and the blue shark *P. glauca*. In turn, *P. glauca* formed a well-supported (bv = 93) clade with *C. acronotus, C. albimarginatus, C. amblyrhynchos, C. falciformis*, and *C. tjutjot*. Within the *Carcharhinus* + *Trianodon* + *Prionace* clade, the newly assembled mitochondrial genome of *C. longimanus* was sister (bv = 100) to a second mitochondrial genome of *C. longimanus* (NC025520). In turn, *C. longimanus* was sister to *C. obscurus* (bv = 100). Most of the internal relationships within the genus *Carcharhinus* were not resolved in our phylogenetic analysis based on translated mitochondrial PCGs. Nonetheless, fully or well supported sister relationships included *Carcharhinus limbatus* + *Carcharhinus amblyrhynchoides* (bv = 100), *C brevipinna* + *C brachyurus* (bv = 97), and *C perezii* + *C sorrah* (bv = 92). In line with Baeza [22] and Winn [41], our results suggest that the genus *Carcharhinus*, among others in the family Carcharhinidae, is in need of systematic re-arrangements. We argue in favor of additional studies assembling mitochondrial genomes in other representatives of this family to resolve internal relationships in the remarkable clade of sharks that is currently experiencing major environmental challenges.

## Conclusion

This study assembled the complete mitochondrial genome of the oceanic whitetip shark, *C. longimanus*, which is considered by the IUCN (International Union for Conservation of Nature) Red List of Threatened Species as “vulnerable” throughout its range and “critically endangered” in the western north Atlantic. This genomic resource can serve as a baseline for biomonitoring and bioprospecting of this epipelagic shark using environmental DNA (eDNA) metabarcoding and/or metagenomic strategies. Also, the assembled mitochondrial genome plus others sequenced for other closely and distantly related species can be used as references to accurately detect the presence of imperilled species in the marketplace and flag mislabeling so to ensure compliance with trade regulations. By employing genomic tracking, the illegal trade of oceanic whitetip sharks and other imperilled species in the marketplace can be minimized. Furthermore, insights into the patterns of selective pressures and their effects on PCGs can enhance our understanding of mitogenome evolution and shed light on broader concepts of adaptation in shark species. Considering the significance of analyzing evolutionary patterns and addressing phylogenetic complexities, we emphasize the need for expanding genomic resources for this and other representatives of the family Carcharhinidae.

## Supplementary Information

Below is the link to the electronic supplementary material.

Supplementary file1 (DOCX 18 KB)

Supplementary file2 (DOCX 14 KB)

Supplementary file3 (PDF 401 KB)

Supplementary file4 (PDF 376 KB)

## Data Availability

The sequence data are part of a project to sequence mitochondrial genomes of marine fishes occurring in the Exclusive Economic Zone of the United States based on voucher specimens (BioProject: PRJNA720393) and data are deposited on GenBank (BioSample: SAMN31811566).

## Abbreviations

IUCN: International Union for Conservation of Nature
NCBI: National Center for Biotechnology Information
rRNA: Ribosomal RNA
tRNA: Transfer RNA
PCGs: Protein coding genes
cox: Cytochrome c oxidase
nad: Nicotinamide adenine dinucleotide
cytb: Cytochrome b
Ka: Number of non-synonymous substitutions per non-synonymous site
Ks: Number of synonymous substitutions per synonymous site
CR: Control region
gDNA: Genomic DNA
ML: Maximum likelihood
