# Supplementary material for: Detailed characterization of the complete mitochondrial genome of the oceanic whitetip shark Carcharhinus longimanus (Poey, 1861)
Source: Mol Biol Rep. 2024 Jul 19;51(1):826. doi: 10.1007/s11033-024-09780-3 (PMC11271432; doi:10.1007/s11033-024-09780-3)
Supplement: Supplementary file 1 — Supplementary file1 (DOCX 18 KB) [file 11033_2024_9780_MOESM1_ESM.docx]

**The mitochondrial genome of the Oceanic Whitetip Shark *Carcharhinus longimanus***

Molecular Biology Reports

Sadia A, Kamal^a^, Katherine E. Bemis^b, c^ J. Antonio Baeza^d,e,f^

^a^ Department of Fisheries Biology and Genetics, Sher-e-Bangla Agricultural University, Dhaka, Bangladesh

^b^ National Systematics Laboratory, Office of Science and Technology, NOAA Fisheries, Washington, DC, USA

^c^ Department of Vertebrate Zoology, National Museum of Natural History, Smithsonian Institution, Washington, D.C, USA

^d^ Department of Biological Sciences, Clemson University, Clemson, SC, USA

^e^ Smithsonian Marine Station at Fort Pierce, Smithsonian Institution, Fort Pierce, FL, USA

^f^ Departamento de Biología Marina, Universidad Catolica del Norte, Coquimbo, Chile

*** Corresponding author.** E-mail address: baeza.antonio@gmail.com (J.A. Baeza). Department of Biological Sciences, Clemson University, Clemson, SC, USA

**Supplementary Table S1.** Codon usage of *Carcharhinus longimanus* mitochondrial protein coding genes.

| Amino Acid | Codon | Number | Frequency (%) | RSCU |
| --- | --- | --- | --- | --- |
| Alanine (A) | GCG  GCA  GCT  GCC | 4  92  94  86 | 0.105  2.414  2.467  2.257 | 0.057971  1.333333  1.362319  1.246377 |
| Cysteine (C) | TGT  TGC | 15  8 | 0.394  0.210 | 1.304348  0.695652 |
| Aspartic acid (D) | GAT  GAC | 42  31 | 1.102  0.813 | 1.150685  0.849315 |
| Glutamic acid (E) | GAG  GAA | 7  91 | 0.184  2.388 | 0.142857  1.857143 |
| Phenylalanine (F) | TTT  TTC | 114  126 | 2.991  3.306 | 0.95  1.05 |
| Glycine (G) | GGG  GGA  GGT  GGC | 12  102  64  50 | 0.315  2.676  1.679  1.312 | 0.210526  1.789474  1.122807  0.877193 |
| Histidine (H) | CAT  CAC | 51  53 | 1.338  1.391 | 0.980769  1.019231 |
| Isoleucine (I) | ATT  ATC | 199  154 | 5.222  4.041 | 1.127479  0.872521 |
| Lysine (K) | AAG AAA | 2  83 | 0.052  2.178 | 0.047059  1.952941 |
| Leucine (L) | TTG  TTA  CTG  CTA  CTT  CTC | 13  202  5  179  129  137 | 0.341  5.3  0.131  4.697  3.385  3.595 | 0.117293  1.822556  0.045113  1.615038  1.16391  1.23609 |
| Methionine (M) | ATG  ATA | 23  139 | 0.604  3.647 | 0.283951  1.716049 |
| Asparagine (N) | AAT AAC | 64  87 | 1.679  2.283 | 0.847682  1.152318 |
| Proline (P) | CCG  CCA  CCT  CCC | 5  102  59  47 | 0.131  2.676  1.548  1.233 | 0.093897  1.915493  1.107981  0.882629 |
| Glutamine (Q) | CAG  CAA | 1  93 | 0.026  2.440 | 0.021277  1.978723 |
| Arginine (R) | CGG CGA CGT  CGC | 2  43  12  16 | 0.052  1.128  0.315  0.420 | 0.109589  2.356164  0.657534  0.876712 |
| Serine (S) | AGT  AGC  TCG  TCA  TCT  TCC | 23  30  2  103  54  55 | 0.604  0.787  0.052  2.703  1.417  1.443 | 0.516854  0.674157  0.044944  2.314607  1.213483  1.235955 |
| Threonine (T) | ACG ACA  ACT ACC | 2  124  75  89 | 0.052  3.254  1.968  2.335 | 0.027586  1.710345  1.034483  1.227586 |
| Valine (V) | GTG GTA GTT GTC | 5  76  52  30 | 0.131  1.994  1.364  2.335 | 0.122699  1.865031  1.276074  0.736196 |
| Tryptophan (W) | TGG TGA | 5  118 | 0.131  3.096 | 0.081301  1.918699 |
| Tyrosine (Y) | TAT TAC | 72  47 | 1.889  1.233 | 1.210084  0.789916 |
| End  End  End  End | AGG  AGA  TAG  TAA | 1  0  3  7 | 0.026  0  0.079  0.184 | 0  0  0  0 |
