# Supplementary material for: Detailed characterization of the complete mitochondrial genome of the oceanic whitetip shark Carcharhinus longimanus (Poey, 1861)
Source: Mol Biol Rep. 2024 Jul 19;51(1):826. doi: 10.1007/s11033-024-09780-3 (PMC11271432; doi:10.1007/s11033-024-09780-3)
Supplement: Supplementary file 2 — Supplementary file2 (DOCX 14 KB) [file 11033_2024_9780_MOESM2_ESM.docx]

**The mitochondrial genome of the Oceanic Whitetip Shark *Carcharhinus longimanus***

Journal: Molecular Biology Reports

Sadia A, Kamal^a^, Katherine E. Bemis^b, c^ J. Antonio Baeza^d,e,f^

^a^ Department of Fisheries Biology and Genetics, Sher-e-Bangla Agricultural University, Dhaka, Bangladesh

^b^ National Systematics Laboratory, Office of Science and Technology, NOAA Fisheries, Washington, DC, USA

^c^ Department of Vertebrate Zoology, National Museum of Natural History, Smithsonian Institution, Washington, D.C, USA

^d^ Department of Biological Sciences, Clemson University, Clemson, SC, USA

^e^ Smithsonian Marine Station at Fort Pierce, Smithsonian Institution, Fort Pierce, FL, USA

^f^ Departamento de Biología Marina, Universidad Catolica del Norte, Coquimbo, Chile

*** Corresponding author.** E-mail address: baeza.antonio@gmail.com (J.A. Baeza). Department of Biological Sciences, Clemson University, Clemson, SC, USA

**Supplementary Table S2.** Position and identity of microsatellite repeats in the control region of *C. longimanus* mitochondrial genome.

| **Position** | **Cycle** | **Repeats** | **Sequence** |
| --- | --- | --- | --- |
| 31 | 2 | 3 | AAAAAA |
| 372 | 2 | 3 | AAAAAA |
| 549 | 2 | 3 | TTTTTT |
| 739 | 2 | 3 | TTTTTT |
| 768 | 2 | 3 | AAAAAA |
| 903 | 2 | 4 | AAAAAAAA |
| 922 | 2 | 5 | AAAAAAAAAA |
| 934 | 2 | 3 | TTTTTT |
| 949 | 2 | 3 | CCCCCC |
| 1034 | 2 | 4 | TATATATA |
