# Supplementary material for: Detailed characterization of the complete mitochondrial genome of the oceanic whitetip shark Carcharhinus longimanus (Poey, 1861)
Source: Mol Biol Rep. 2024 Jul 19;51(1):826. doi: 10.1007/s11033-024-09780-3 (PMC11271432; doi:10.1007/s11033-024-09780-3)
Supplement: Supplementary file 3 — Supplementary file3 (PDF 401 KB) [file 11033_2024_9780_MOESM3_ESM.pdf]

Supplementary Material

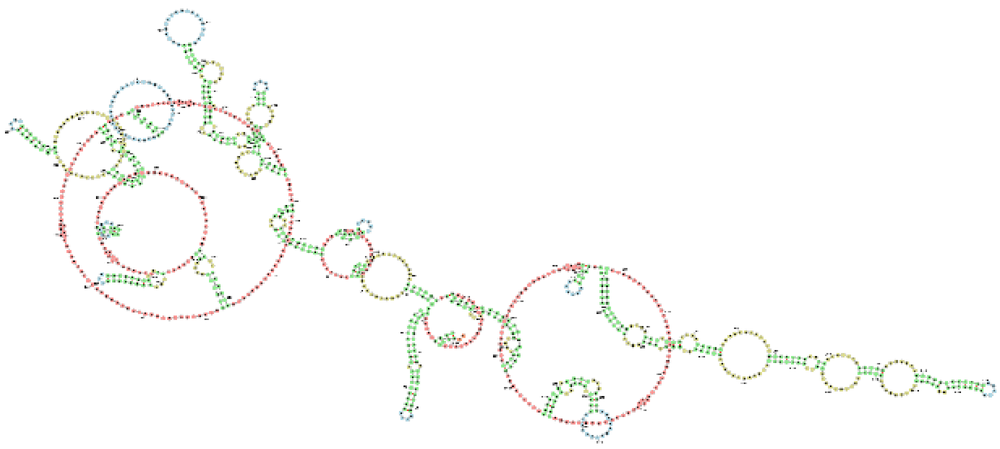

(a)

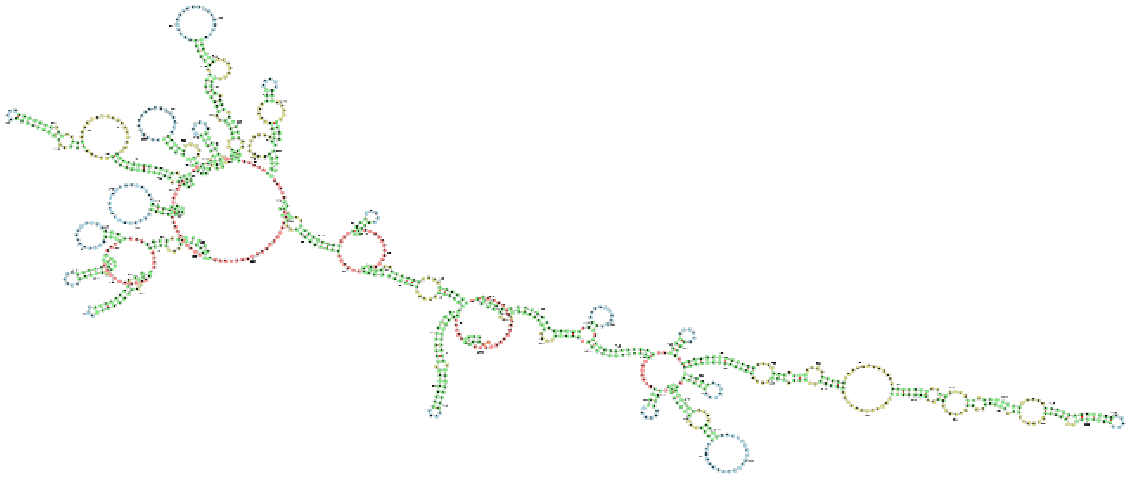

(b)

Supplementary Fig 1. (a) Optimal and (b) Centroid secondary structures of Control region predicted by RNAfold.
